# BioArchLinux: community-driven fresh reproducible software repository for life sciences

**DOI:** 10.1093/bioinformatics/btaf106

**Published:** 2025-03-11

**Authors:** Guoyi Zhang, Pekka Ristola, Han Su, Bipin Kumar, Boyu Zhang, Yujin Hu, Michael G Elliot, Viktor Drobot, Jie Zhu, Jens Staal, Martin Larralde, Shun Wang, Yun Yi, Haoran Yu

**Affiliations:** Evolution & Ecology Research Centre, School of Biological, Earth, Environmental Sciences, UNSW Sydney, Sydney, NSW 2052, Australia; Australian Museum Research Institute, Australian Museum, 1 William St, Sydney, NSW 2010, Australia; BioArchLinux Member, Helsinki, 00100, Finland; BioArchLinux Member, Beijing, 100000, China; Obront Biotech Pvt. Ltd., Hyderabad, 500007, India; Beijing Forestry University, Beijing, 100083, China; Shenzhen Research Institute of Big Data, Shenzhen, 518172, China; Department of Evolution, University of Groningen, Groningen, 9700 AB, The Netherlands; Pharm InnTech LLC, Moscow, 127299, Russia; Microbiota I-Center (MagIC), Hong Kong SAR 999077, China; Li Ka Shing Institute of Health Sciences, Faculty of Medicine, The Chinese University of Hong Kong, Hong Kong SAR 999077, China; Department of Biomedical Molecular Biology, Ghent University, 9000, Belgium; Leiden University Center for Infectious Diseases, Leiden University Medical Center (LUMC), Leiden, 2300 RA, The Netherlands; Structural and Computational Biology Unit, EMBL, Heidelberg, 69117, Germany; JASP Team Member, Shenzhen, 518000, China; BioArchLinux Member, Beijing, 100000, China; Xi’an University, Xi’an, 710065, China

## Abstract

**Motivation:**

The BioArchLinux project was initiated to address challenges in bioinformatics software reproducibility and freshness. Relying on Arch Linux's user-driven ecosystem, we aim to create a comprehensive and continuously updated repository for life sciences research.

**Results:**

BioArchLinux provides a PKGBUILD-based system for seamless software packaging and maintenance, enabling users to access the latest bioinformatics tools across multiple programming languages. The repository includes Docker images, Windows Subsystem for Linux (WSL) support, and Junest for nonroot environments, enhancing accessibility across platforms. Although being developed and maintained by a small core team, BioArchLinux is a fast-growing bioinformatics repository that offers a participatory and community-driven environment.

**Availability and implementation:**

The repository, documentation, and tools are freely available at https://bioarchlinux.org and https://github.com/BioArchLinux. Users and developers are encouraged to contribute and expand this open-source initiative.

## 1 Introduction

Bioinformatics software and the development of new algorithms and tools are crucial for analyzing and interpreting biological data (Hettrick 2014), enabling researchers to make significant discoveries in genomics, proteomics, and other fields (Horner *et al.* 2010) by facilitating the processing of large datasets and visualization of complex biological processes. One concern with an increased reliance on complex software in data processing is the transparency of the methods used, and the reproducibility of the processes used for the analysis (Waltemath and Wolkenhauer 2016, Kanterakis *et al.* 2019, Raghupathi *et al.* 2022). Reproducibility ensures that conclusions from scientific experiments and analyses can be trusted, and it is essential for validating results and building upon previous work (Baker 2016). However, when it comes to bioinformatics software, reproducibility is related to a multitude of factors (Baykal *et al.* 2024), in particular the chosen platform.

Linux is a major bioinformatics analysis platform known for its flexibility and modularity (Yoo 2016). However, this also has a problem: distribution community fragmentation. Fragmented communities have different compilation processes, and compilation is an important factor affecting the repeatability of bioinformatics software (Grüning *et al.* 2018). The distribution’s “software freshness” (i.e. packages are at the latest official release) affects whether users can use the most up-to-date software and the distribution’s software coverage, reflecting the availability of pre-compiled software in repositories. Those two factors will influence whether users choose to compile software by themselves or use pre-compiled software provided by repositories.

Users often choose to compile software themselves when the community-provided software is insufficient or outdated. Facing the significant challenges of bioinformatics software on repeatability and freshness, Arch Linux excels in providing the latest software and an easy-to-use functional shell script packing system, making it an ideal mainstream distribution for freshness (Legay *et al.* 2021, Repology Team 2024) and easy user-driven contribution that ensures a high software coverage (e.g. ArchLinux User Repository is the second largest Linux user-contributed repository based on repology statistics due to Arch Linux package style). Considering these factors, we initiated the BioArchLinux project based on the Arch Linux environment to address these issues to provide a more efficient solution to the problem of software freshness and coverage in bioinformatics.

## 2 Implementation

BioArchLinux brings packages that are also offered by other bioinformatics repositories, covering a wide range of tools—from traditional morphology analysis software developed over the past 30 years to state-of-the-art genomics analysis tools. Our platform is designed to run natively on x86 bare metal and supports macOS via Docker, Windows via WSL, and Linux HPC environments, where installations can be executed without root privileges using Junest.

**Figure 1.**
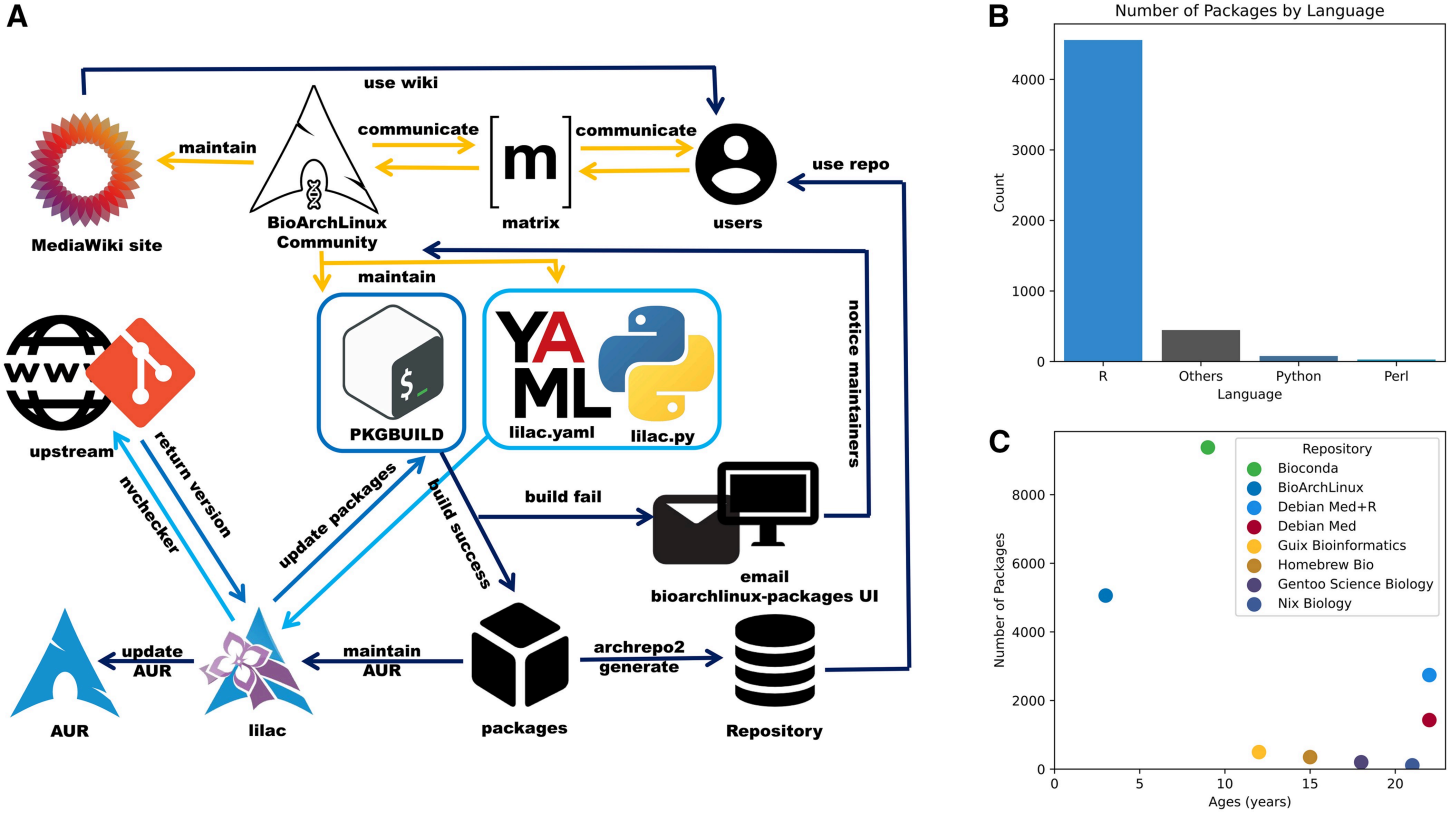
Workflow and statistics of BioArchLinux. (A) Workflow of BioArchLinux community. (B) Number of BioArchLinux packages by programming language (October 2024). (C) Current number of packages and age of different repositories (October 2024).

We use a PKGBUILD-based system to create and manage packages (Fig. 1A). Shell scripts are familiar and widely used, and their function-based structure allows for a clear definition of different packaging processes such as preparation, compilation, and packaging. Our packaging tool, lilac, can handle lilac.yaml and lilac.py scripts facilitating easy interaction with Python for version updates. This allows a team of around 15 people to maintain over 5000 packages as well as developing and maintaining development software. Although many of the packages we maintain are R packages, which are relatively easier to manage and which significantly contribute to the growth of our repository, we currently maintain around 500 non-R packages.

Given the complexity of downstream dependencies, we ensure reproducibility by providing container images, such as Docker and Junest, that encapsulate the entire environment, enabling analyses to be consistently replicated. In doing so, our Docker images offer a consistent and isolated platform for running bioinformatics software on macOS, Windows, and other Linux distributions. Fresh Docker images are provided daily to ensure users always have access to the latest software, usually about 200 MB. Junest (Squillace 2024) provides a lightweight, nonroot solution for running Arch Linux packages on other Linux distributions, making it accessible to a broader range of nonroot users. For Windows users, we support the Windows Subsystem for Linux (WSL), allowing them to run Linux-based bioinformatics tools natively on Windows 10 and above.

Pacman 7.0 introduces new features such as sandboxing and DownloadUser, enhancing security and user experience by isolating package installations and managing downloads more efficiently. Our Git repository also offers excellent tracking capabilities to ensure repeatability.

Although Arch Linux provides a snapshot website to help users downgrade and roll back, we currently lack the resources to offer this functionality ourselves. Users can create the environment using Arch Linux archive snapshots and rebuild packages using our git repository to achieve reproducibility. Once additional computing resources become available, we aim to provide snapshot capabilities to further enhance reproducibility.

For some of our maintained packages, such as beagle-libs, IQ-TREE, MrBayes, and Eugene, we offer various specialized optimization versions of pre-compiled packages. These include optimizations utilizing GPU (OpenCL/CUDA) technologies and parallelization. These optimizations cater to different user needs and deliver enhanced performance. Providing more optimization options to users will always be our future aim.

To better serve the whole Linux software ecology instead of only our maintained repository, our collaboration with upstream and Arch Linux maintainers has been a cornerstone of our efforts, including the regular submission of patches upstream. Given that Arch Linux is an aggressively updated distribution, we are often the first community to identify and address source code and latest version compiler incompatibility issues, e.g. GCC. Our contributions span a variety of packages, including RevBayes, TrinityRNAseq, Augustus, eugene, BEAST-mcmc, Open Delta, and more.

In addition to package maintenance, we have developed several tools to aid in the upkeep of Arch Linux-based distributions and projects. We provide a comprehensive package search page to help users find relevant information easily. We have also co-contributed the development of the lilac building tool, which has proven invaluable for other Arch Linux-based distributions. Furthermore, the nvchecker tool, which we use and contribute to, has been widely adopted by many Linux distributions, underscoring our commitment to enhancing the broader Linux ecosystem.

## 3 Results

Our repositories contain R, Python, Perl, and other programming language packages, e.g. C/C++ (Fig. 1B), which breaks the limitation of specific language package managers. We are not the sole initiative distributing bioinformatics software, there are also other repositories available (Fig. 1C). Though we only have 15 maintainers, compared to Bioconda’s large team, our repository packages number is more than half of Bioconda’s (Fig. 1C). However, this difference is attributed to the distinct development and maintenance philosophies among different Linux distributions.

Crucially, our project encourages participation, whether it is for maintaining existing software or releasing new packages. We strive to maintain a high level of freshness and aim to create a comprehensive, language-agnostic, and easy-to-install collection of life sciences software. We welcome everyone to join our community and contribute to the BioArchLinux project. Together, we can create a robust and user-friendly software repository for the life sciences.
